# Gut-Derived Metabolite Indole-3-Propionic Acid Modulates Mitochondrial Function in Cardiomyocytes and Alters Cardiac Function

**DOI:** 10.3389/fmed.2021.648259

**Published:** 2021-03-22

**Authors:** Maren Gesper, Alena B. H. Nonnast, Nina Kumowski, Robert Stoehr, Katharina Schuett, Nikolaus Marx, Ben A. Kappel

**Affiliations:** Department of Internal Medicine 1, University Hospital Aachen, Rheinisch-Westfälische Technische Hochschule (RWTH) Aachen University, Aachen, Germany

**Keywords:** gut microbiota, microbial metabolites, heart failure, mitochondrial dysfunction, cardiomyocyte physiology

## Abstract

**Background:** The gut microbiome has been linked to the onset of cardiometabolic diseases, in part facilitated through gut microbiota-dependent metabolites such as trimethylamine-*N*-oxide. However, molecular pathways associated to heart failure mediated by microbial metabolites remain largely elusive. Mitochondria play a pivotal role in cellular energy metabolism and mitochondrial dysfunction has been associated to heart failure pathogenesis. Aim of the current study was to evaluate the impact of gut-derived metabolites on mitochondrial function in cardiomyocytes via an *in vitro* screening approach.

**Methods:** Based on a systematic Medline research, 25 microbial metabolites were identified and screened for their metabolic impact with a focus on mitochondrial respiration in HL-1 cardiomyocytes. Oxygen consumption rate in response to different modulators of the respiratory chain were measured by a live-cell metabolic assay platform. For one of the identified metabolites, indole-3-propionic acid, studies on specific mitochondrial complexes, cytochrome c, fatty acid oxidation, mitochondrial membrane potential, and reactive oxygen species production were performed. Mitochondrial function in response to this metabolite was further tested in human hepatic and endothelial cells. Additionally, the effect of indole-3-propionic acid on cardiac function was studied in isolated perfused hearts of C57BL/6J mice.

**Results:** Among the metabolites examined, microbial tryptophan derivative indole-3-propionic acid could be identified as a modulator of mitochondrial function in cardiomyocytes. While acute treatment induced enhancement of maximal mitochondrial respiration (+21.5 ± 7.8%, *p* < 0.05), chronic exposure led to mitochondrial dysfunction (−18.9 ± 9.1%; *p* < 0.001) in cardiomyocytes. The latter effect of indole-3-propionic acids could also be observed in human hepatic and endothelial cells. In isolated perfused mouse hearts, indole-3-propionic acid was dose-dependently able to improve cardiac contractility from +26.8 ± 11.6% (*p* < 0.05) at 1 μM up to +93.6 ± 14.4% (*p* < 0.001) at 100 μM. Our mechanistic studies on indole-3-propionic acids suggest potential involvement of fatty acid oxidation in HL-1 cardiomyocytes.

**Conclusion:** Our data indicate a direct impact of microbial metabolites on cardiac physiology. Gut-derived metabolite indole-3-propionic acid was identified as mitochondrial modulator in cardiomyocytes and altered cardiac function in an *ex vivo* mouse model.

## Introduction

Heart failure (HF) is a multifactorial syndrome characterized by mechanical cardiac dysfunction and inability to provide sufficient perfusion to the body. Despite recent therapeutic advances, morbidity and mortality of HF still remains high. Therefore, there is an urgent need to identify HF-related pathomechanisms to develop novel preventive and therapeutic strategies. During the past decade, gut microbiota have been identified as important contributor to various pathologic conditions including cardiovascular disease (CVD) (1). While gut microbiota are able to influence the host in different ways, technical advances in metabolomics identified gut-derived metabolites as mediators of CVD and other metabolic diseases. These metabolites enter the blood flow and are thereby able to interact with cells of the cardiovascular system (1, 2). One prominent microbial metabolite is trimethylamine-*N*-oxide (TMAO). As part of bacterial choline metabolism, TMAO has been associated with progression of CVD and cardiovascular mortality (3–5). Besides TMAO, other microbial metabolites, like short-chain fatty acids (SCFA) and tryptophan derivatives, have been linked to cardiovascular disorders (6, 7).

While past studies have focused on gut microbiota-related mechanism associated to atherosclerosis, atherothrombosis and myocardial infarction (4, 8, 9), only few have investigated the role of gut bacteria on HF. Patients with HF exhibit changes in the gut flora (10, 11). However, pathomechanisms between intestinal bacteria and HF remain elusive.

Mitochondria play a pivotal role in metabolism and bioenergetics, particularly in an organ with high energy demand, such as the heart. Mitochondrial dysfunction is a characteristic of HF and one of the first steps during metabolic remodeling in the failing heart (12–15). Although a vast number of circulating microbiota-derived metabolites have been described, their impact on cardiac metabolism is unknown. Therefore, we conducted this study to screen the influence of microbial metabolites on metabolic function in cardiomyocytes. Using a live-cell metabolic assay platform with focus on mitochondrial respiration, 25 circulating gut-derived metabolites of seven different pathways were tested in HL-1 cardiomyocytes. Here, we identified indole-3-propionic acid (IPA), a microbial tryptophan derivative, as modulator of mitochondrial function *in vitro*. Using an isolated perfused heart mouse model, we were able to show that IPA exhibits direct effect on cardiac function.

Together, our data suggest that gut microbiota are capable to impact cardiac physiology via gut-derived metabolites. Our data may therefore provide implications for further studies investigating the role of gut microbiota on HF.

## Materials and Methods

### Systematic Medline Search

To identify and select different gut-derived metabolites, a systematic Medline search was performed (July 2017). The following search term was used: [((bacteria[Title] OR bacterial[Title] OR flora[Title] OR microbiota[Title] OR microbiome[Title] OR microbe[Title] OR microflora[Title])) AND (gut[Title] OR intestine[Title] OR intestinal[Title] OR colon[Title] OR colonic[Title] OR cecum[Title] OR caecum[Title] OR cecal[Title] OR caecal[Title] OR stool[Title])) AND (metabolite[Title/Abstract] OR metabolites[Title/Abstract] OR metabolome[Title/Abstract] OR metabolomics[Title/Abstract] OR metabolic[Title/Abstract]]. Based on these results, 25 commercially available metabolites were selected with potential biological function on cardiovascular or other biological systems.

### Reagents

If not differently specified, all reagents and chemicals were purchased from Sigma-Aldrich (St. Louis, MO, USA).

### Metabolites Used for Cell Experiments

Trimetyhlamine (TMA), trimethylamine-*N*-oxide (TMAO), phenylacetylglutamine (Bachem; Bubendorf, Switzerland), 3,4-dihydroxyphenylacetic acid, 4-hydroxyphenylacetic acid, sodium taurodeoxycholate hydrate, sodium propionate and sodium butyrate were dissolved in distillated water. Indole-3-propionic acid (IPA) was also dissolved as aqueous solution and adjusted to pH: 7.4. Working solutions were prepared in distillated water. Indole, indole-3-acetic acid, 2-oxindole, phenylacetic acid, p-cresol, trans-3-hydroxycinnamic acid, 3-(4-hydroxyphenyl)propionic acid, benzoic acid, 3-hydroxybenzoic acid, hippuric acid, o-hydroxyhippuric acid, cholic acid, and valeric acid were dissolved in dimethyl sulfoxide (DMSO). Deoxycholic acid and ursodeoxycholic acid were dissolved in 100% ethanol (Carl Roth; Karlsruhe, Germany). It was ensured that final concentrations of vehicles (ethanol/DMSO) did not exceed 0.1% of the total volume.

### Cell Culture

For the metabolic screening of the metabolites three different cell types were used. Murine cardiomyocytes (HL-1) (16) were cultured in Claycomb medium supplemented with 10% (v/v) fetal bovine serum, 1% (v/v) penicillin/streptomycin (100 Units/ml penicillin and 100 μg/ml streptomycin), 0.1 mM norepinephrine (in 30 mM ascorbic acid) and 2 mM L-glutamine. Flasks, 96-well and Seahorse XF96 cell culture plates (Agilent; Santa Clara, CA, USA) were pre-coated with 0.02% (v/v) gelatin containing 5 μg/ml fibronectin.

Human hepatoma cell line (Huh7) (17) was cultured in Dulbecco's Modified Eagle's Medium (DMEM—high glucose) supplemented with 10% (v/v) fetal bovine serum and 1% (v/v) penicillin/streptomycin (100 Units/ml penicillin and 100 μg/ml streptomycin).

Human umbilical vein endothelial cells (HUVEC) (18) were cultured in Endothelial Cell Growth Medium-2 (EGM-2; Lonza, Basel, Switzerland). HUVECs were used between passage 3 and 8. All cell types were cultured at 37°C in a humidified atmosphere with 5% CO_2_.

### Cell Viability Assay

To analyze the cell viability in response to the metabolites, a resazurin-based assay with the reagent PrestoBlue (ThermoFisher; Walthman, MA, USA) was used (19). The assay was performed according to the manufacture's protocol. Briefly, cells were seeded with a density of 10,000 cells/well in 96-well-plates (Corning 3610; Corning, NY, USA). Cells were treated with different concentrations of the metabolites (1 μM−1 mM) for 24 h at 37°C (with 5% CO_2_). After the treatment, cells were incubated with media containing 1× PrestoBlue for 1 h at 37°C (with 5% CO_2_) and fluorescence intensity was measured at Ex/Em 560/590 nm (Tecan infinite M200; Tecan Group Ltd., Männedorf, Switzerland).

### Cell Proliferation Assay

Cell proliferation in response to indole-3-propionic acid (IPA) treatment was detected by incorporation of the pyrimidine analog 5-Bromo-2′-deoxyuridine (BrdU) into the DNA (20). The cell proliferation ELISA, BrdU (chemiluminescence) (Roche; Basel, Switzerland) was used according to the manufacture's protocol. Cells were seeded with a density of 2,000 cells/well in 96-well-plates and treated with different concentrations of IPA (10 μM−1 mM). Cells were incubated at 37°C (with 5% CO_2_) and medium was changed daily. Incorporation of BrdU into the DNA was measured via luminescence with an integration time of 2,500 ms (Tecan infinite M200). Proliferation via BrDU assay was measured after 24 h, 48 h, and 72 h.

### Real-Time Extracellular Flux Analysis

Metabolic function in cells in response to the metabolites was characterized by Seahorse XFe96 Flux Analyzer (Agilent). This technique allows real-time measurements of the oxygen consumption rate (OCR) and glycolysis (ECAR) in living cells. Two different Seahorse Kits, Seahorse XF Cell Energy Phenotype Test and Seahorse XF Mito Stress Test, were used to characterize metabolic function of cells in response to gut-derived metabolites.

Cells were seeded with a density of 10,000 cells/well on XF96 cell culture plates and treated with metabolites for 24 h (37°C, 5% CO_2_). In selected experiments, cells were treated with IPA for shorter periods of time. On the day of the assay, medium was changed to unbuffered XF Assay Medium (Agilent) (supplemented with 10 mM D-glucose, 2 mM L-glutamine and 1 mM sodium pyruvate; adjusted to pH: 7,4) to a final volume of 180 μl/well, after three washing steps with assay medium. For pre-equilibration, cells were incubated for 1 h in a non-CO_2_ incubator at 37°C. In addition, one day before the assay, XFe96 Sensor Cartridge (Agilent) was hydrated with autoclaved water over night at 37°C in a non-CO_2_ atmosphere. Before the Sensor Cartridge was loaded with different inhibitors to the respiration chain, water was exchanged with Seahorse XF Calibrant Solution (Agilent) and incubated for 45 min at 37°C (non-CO_2_).

For the Seahorse XF Cell Energy Phenotype Test, ATP synthase inhibitor oligomycin at a final concentration of 1 μM and the uncoupler carbonyl cyanide-*4-*(trifluoromethoxy)phenylhydrazone (FCCP) at a final concentration of 0.4 μM were injected at the same time point after a general baseline measurement of OCR.

For the Seahorse Mito Stress Kit, oligomycin (1 μM), FCCP (0.4 μM) and a mixture of rotenone/antimycin A (final concentration of mix: 0.5 μM), inhibitors of complex I and II, were injected sequentially after baseline measurements.

The general protocol of the measurements includes three baseline measurements with mix (3 min)/measure (3 min) followed by injection of port A. Afterwards respiration was measured for five (Cell Energy Phenotype) to three times (Mito Stress) with mix (3 min)/measure (3 min). For the Mito Stress, port B and C was injected subsequently with the same measurement cycle of three times mix/measure in between.

### Analysis of Respiratory Activity in Permeabilized Cells

Measurement of mitochondrial respiration in permeabilized cells by Seahorse XFe96 Flux Analyzer allows a detail overview on the different complexes of mitochondrial respiratory chain and their activity (21, 22). For this, HL-1 cardiomyocytes were seeded in a density of 10,000 cells/well on XF96 cell culture plates and were treated with IPA for 24 h (37°C, 5% CO_2_). Before the measurement, cells were washed with 1× mannitol and sucrose (MAS) buffer (220 mM mannitol, 70 mM sucrose, 10 mM KH_2_PO_4_, 5 mM MgCl_2_, 2 mM HEPES, and 1 mM EGTA). After a washing step, 1× MAS buffer was changed to 1× MAS buffer supplemented with 4 mM ADP (ADP sodium salt) and 10 μg/ml saponin, as permeabilizing agent, in a final volume of 180 μl/well. Saponin only permeabilizes the plasma membrane without affecting mitochondrial membrane (21), thus enabling the addition of different substrates to the complexes of the respiratory chain.

To characterize the activity of the different complexes sequentially, 10 mM pyruvate and 1 mM malate were further added in 1× MAS buffer to analyze complex I of the respiratory chain. For inhibition of complex I, 20 μM rotenone (final concentration in well: 2 μM) was injected via port A. Subsequently, 100 mM succinate (final concentration: 10 mM) was injected to analyze complex II activity. To inhibit complex II, 20 μM antimycin A (final concentration: 20 μM) was injected via port C. At the end of the measurement, complex IV was characterized by the injection of 1 mM *N,N,N*′*,N*′-Tetrametyhl-*p*-phenylenediamine (TMPD) and 100 mM ascorbic acid (final concentrations: 0.1/10 mM). Measurement periods were shorter with cycles of two times mix (0.5 min)/wait (0.5 min)/measure (2 min) without equilibration step.

Beside the comprehensive study of the respiratory complexes, respiratory activity based on medium- and long-chain fatty acid oxidation was measured. Therefore, 1× MAS buffer was supplemented with 4 mM ADP, 10 μg/ml saponin and either 40 μM palmitoyl-L-carnitine/1 mM malate or 40 μM octanoyl-L-carnitine/1 mM malate. For these experiments, the Mito Stress Kit was performed as described previously with the adapted protocol for permeabilized cells.

### Supplementation of Cytochrome c in Permeabilized Cells

Cytochrome c is an important protein of the respiratory chain and loss can lead to reduction in respiratory activity. Exogenous cytochrome c addition may rescue mitochondrial function (23, 24). Therefore, permeabilized HL-1 cardiomyocytes were measured via Seahorse XFe96 Flux Analyzer after pre-incubation with IPA for 24 h as previously described. 1 mM exogenous cytochrome c (cytochrome c from equine heart; final concentration: 100 μM) was simultaneously injected with saponin (final concentration of 250 μg/ml), 4 mM ADP, 10 mM succinate, and 2 μM rotenone. After two baseline measurements, respiratory activity was measured five times with cycles of mix (0.5 min)/wait (0.5 min)/measure (2 min). At the end of the measurement a mix of 20 μM rotenone/antimycin A (final concentration: 2 μM) was injected to the cells.

### Measurement of Mitochondrial Membrane Potential

To detect direct effects on mitochondrial membrane potential, the fluorescence dye tetramethylrhodamine ethyl ester perchlorate (TMRE) was used. TMRE is a cell permeable, cationic dye, which accumulates to active, negatively-charged mitochondrial membrane (25, 26). This allows a live cell analysis of the membrane potential in response to treatment with metabolites. HL-1 cardiomyocytes were seeded with a density of 10,000 cells/well in 96-well-plates and treated with different concentrations of IPA for 24 h. As positive control, 20 μM FCCP was added 10 min before staining. After pre-incubation with IPA/FCCP, cells were then stained with 200 nM TMRE in media for 30 min (37°C, 5% CO_2_, dark environment). Before measurement of the fluorescence intensity [Ex/Em 540/595 nm (Tecan infinite M200)], cells were washed twice with pre-warmed PBS.

### Measurement of Hydroxyl Radicals

Mitochondrial dysfunction can be caused by reactive oxidative species (ROS). To evaluate ROS production in response to IPA treatment, the dye 2′,7′-dichlorodihydrofluorescein diacetate (H2DCFDA) was used. H2DCFDA is a cell permeable, non-fluorescent dye, which become fluorescent after oxidation with hydroxyl radicals (27). HL-1 cardiomyocytes were seeded in a density of 10,000 cells/well in 96-well-plates and treated with IPA for different time periods (30 min and 24 h). As positive control, 1 mM iron(II) sulfate heptahydrate for 30 min before staining was used. After treatment, cells were washed once with pre-warmed PBS and then stained with 25 μM H2DCFDA in PBS for 30 min (37°C, 5% CO_2_, dark environment). After a second washing step with PBS, fluorescence intensity was measured at Ex/Em 504/529 nm (Tecan infinite M200).

### Langendorff Mouse Model

To characterize whether IPA exhibits direct effects on cardiac function, the *ex vivo* Langendorff mouse model was used (28–30). Animal experiments were approved by the government of North Rhine-Westphalia, Germany. C57BL/6J male mice were purchased from Janvier Labs (Le Genest-Saint-Isle, France). Mice were housed in filtertop cages in a controlled environment (12 h daylight cycle) at the animal facility of the RWTH Aachen University. Mice had free access to water and food. At age of 9–12 weeks (average weight of 22 g) mice were anesthetized with ketamine hydrochloride and xylazine hydrochloride and anticoagulated with 1,000 IE heparin.

The heart was quickly excised and was prepared in ice-cold Krebs–Henseleit buffer. The aorta was cannulated for retrograde perfusion at constant pressure of 90 mmHg using a Langendorff perfusion apparatus (Harvard Apparatus, Massachusetts, USA). The perfusion solution was heated to 37°C and oxygenated with 100% oxygen. A latex balloon filled with distillated water connected to a pressure transducer was inserted into the left ventricle (LV). LV diastolic pressure was set to 12 mmHg. The heart was stimulated resulting in a heart rate of 600 bpm. LV systolic (LVPsys) and LV developed pressure (LVPdp), as well as maximum rate of increase (dLVPmax) and maximum rate of decrease in left ventricular pressure during isovolumic contraction (dLVPmin) were measured continuously (all 2 s) with ISOHEART Isolated Heart Data Acquisition Software (Harvard Apparatus, Massachusetts, USA). Hearts were perfused for a 15 min equilibration period. Following, 200 μL Krebs-Henseleit buffer was administered through the aortic cannula into the heart. After 10 min, dobutamine was administered (1 mg/ml, diluted 1:256). In cycles of 10 min, IPA in different concentrations (1–100 μM) was administered following dobutamine as positive control to ensure proper heart function. Therefore, IPA was dissolved in Krebs-Henseleit buffer and pH-adjusted to 7.4 afterwards. pH-adjusted buffer was used as control to exclude side-effects dependent on buffer administration. In between, washing steps with buffer were performed.

### Statistics

Results are presented as mean ± SEM or SD. For statistical analysis GraphPad Prism version 8 (GraphPad software Inc., San Diego, CA, USA) was used. Data were analyzed by one-way ANOVA or two-way ANOVA with *post-hoc* test. A *p* < 0.05 was considered to be statistically significant. All data are representatives of at least two independent biological replicates. For every measurement four to five technical replicates per group were performed.

## Results

### *In vitro*-Screening of Gut-Derived Metabolites on Metabolic Function in Cardiomyocytes

**Figure 1:**
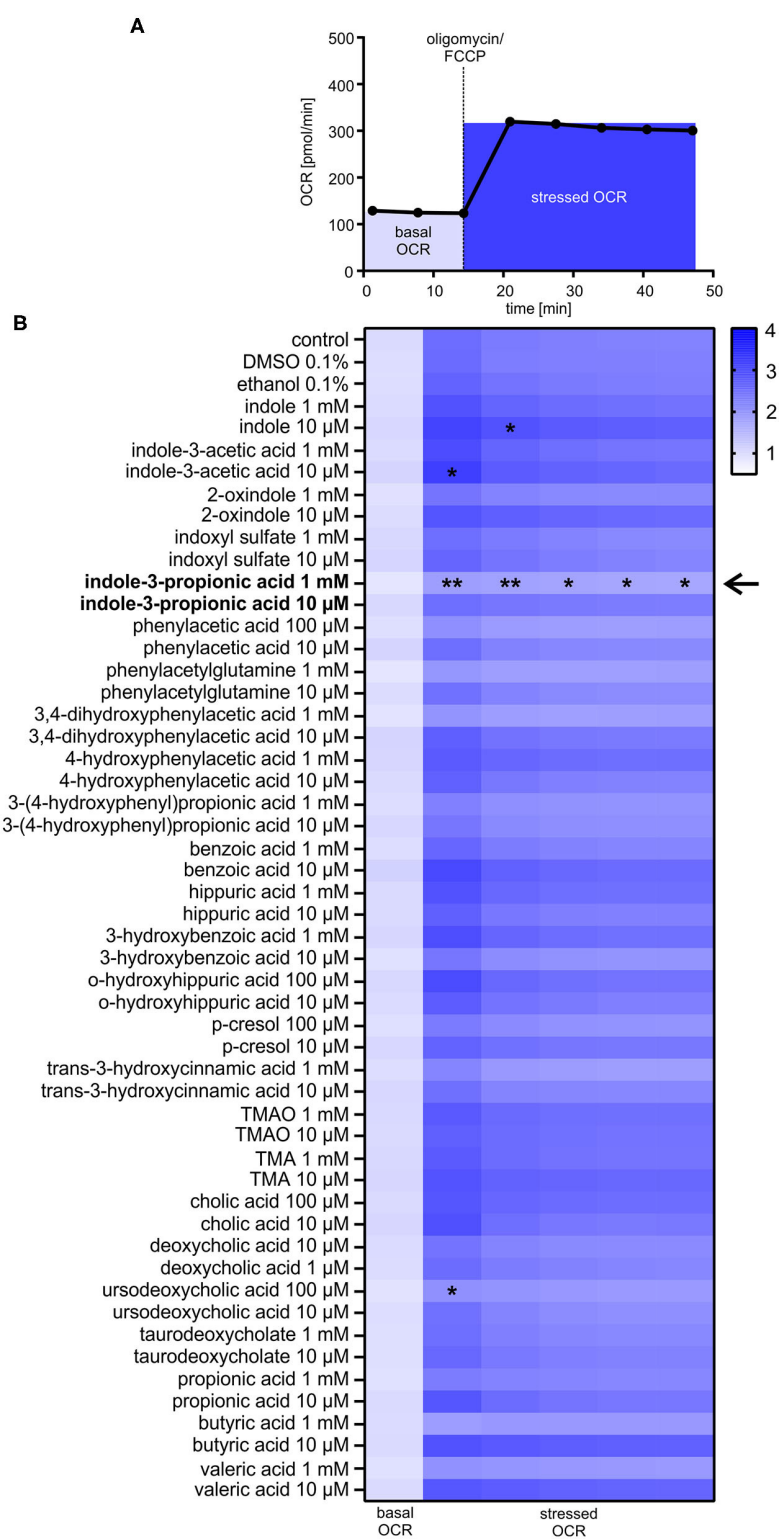
Effects of gut-derived metabolites on mitochondrial respiration in HL-1 cardiomyocytes. **(A**) Flowchart of mitochondrial stress testing by Seahorse Flux Analyzer. Oligomycin and FCCP were simultaneously added to the cells (stressed OCR). **(B)** Effects of gut-derived metabolites on mitochondrial function in HL-1 cardiomyocytes after 24 h incubation with substances. Basal oxygen consumption rate (OCR) of control cells was defined as 1.0. Basal OCR is the sum of three baseline measurements. All metabolites were displayed in relation to control or their vehicle (DMSO/ethanol). All results were verified in two independent replicates with *N* = 5/group. Data were shown as mean. *p < 0.05 and **p < 0.01 by 2-way ANOVA with Dunnett *post-hoc* test. The black arrow indicates the most prominent effect on mitochondrial function of tested metabolites.

Based on a detailed Medline research, 25 commercially available gut-derived metabolites with potential effects on cardiovascular system were selected to analyze their impact on metabolic function in HL-1 cardiomyocytes. Metabolites were categorized into seven different pathways, i.e., microbial tryptophan and choline metabolism (Table 1). Cytotoxity and cell viability in response to metabolites were evaluated in all cell types used (*data not shown*). Only non-toxic concentrations were used for further analyses. To evaluate the effects of gut-derived metabolites on mitochondrial function, we performed mitochondrial stress testing in HL-1 cardiomyocytes. Unexpectedly, we did not observe significant effects on mitochondrial function by most of the tested metabolites (Figures 1A,B). However, we noticed that several microbiota-derived tryptophan metabolites exerted effects on stressed OCR. Particularly, these were indole, indole-3-acetic acid, and indole-3-propionic acid (IPA). IPA exhibited the strongest effect with a clear reduction of −26.8 ± 6.0% of stressed OCR at a concentration of 1 mM (Figure 1B). In contrast to IPA, indole, indole-3-acetic acid and also bile acid ursodeoxycholic acid showed only very moderate effects on stressed OCR (Figure 1B and Supplementary Figure 1).

**Table 1:** Gut-derived metabolites and their impact on host metabolism.

**Metabolism**	**Metabolite**	**Findings in human cohorts**	**Biological mechanisms**
Tryptophan metabolism	Indole	- Negatively associated with atherosclerosis (8)	- Ligand of pregnane X receptor (31)
	Indole-3-acetic acid	- uremic toxin in chronic kidney disease and associated with CVD (32)	- Induces endothelial inflammation and oxidative stress (32) - Ligand of aryl hydrocarbon receptor (33)
	2-oxindole		- Induces hypotension (in rats) (34)
	Indoxyl sulfate	- Uremic toxin in chronic kidney disease and associated with CVD (35) - Associated with aortic calcification and vascular stiffness (35)	- Induction of ROS production (36) - Ligand of aryl hydrocarbon receptor (33) - Associated with cardiac fibrosis and cardiomyocyte hypertrophy (37) - Stimulation of vascular smooth muscle cells proliferation (38) - Induction of inflammatory reactions (39)
	Indole-3-propionic acid (IPA)	- Negatively associated with advanced atherosclerosis (8) - Beneficial effect on lowering type 2 diabetes (40, 41) - Protective effects on development of chronic kidney disease (42)	- Ligand of pregnane X receptor (31) - Antioxidant without pro-oxidative abilities (43, 44) - Inhibition of breast cancer cell proliferation (45)
Phenylalanine metabolism	Phenylacetic acid	- Uremic toxin in chronic kidney disease (46)	- Induction of ROS production (47)
	Phenylacetylglutamine	- association with CVD in chronic kidney disease (48)	- Accelerates platelet clot formation and pro-thrombotic potential (49)
Tyrosine metabolism	3,4-dihydroxyphenylacetic acid		- Increased nitric oxide production (50) - Antioxidative effects (50)
	*4-hydroxyphenylacetic acid*		
	*3-(4-hydroxyphenyl) propionic acid*		
Polyphenols/benzoic acids	*Benzoic acid*		
	Hippuric acid	-Uremic toxin in chronic kidney disease (51) - Correlates with post-operative adverse cardiac events and with MACE in patients with advanced atherosclerosis (8) - Associated with left ventricular hypertrophy on dialysis patients (52)	
	3-Hydroxybenzoic acid	- Prevention of diabetes and coronary heart disease (53)	- Antioxidant effects and scavengers of reactive nitrogen species (53)
	*o-hydroxyhippuric acid*		
	p-cresol	- Uremic toxin in chronic kidney disease associated with CVD (54) - Potential predictor of cardiovascular events (55)	- Dysfunction of endothelial barrier function *in vitro* (56) - Decrease in endothelial proliferation and wound repair (57)
	*Trans-3-hydroxycinnamic acid*		
Choline metabolism	Trimethylamine (TMA)	- Increase of TMA-producing enzymes in ACVD patients (58)	
	Trimethylamine-*N*-oxide (TMAO)	- Associated with atherosclerosis, CVD progression and major cardiovascular events (1)	- Induction of platelet activation and pro-thrombotic (4) - Pro-atherogenic effects (59) - Endothelial dysfunction (60) - Downregulation of anti-inflammatory IL-10 (61)
Bile acids	Cholic acid	- Decreased basal cardiac contractility (62)	
	Deoxycholic acid	- Associated with higher coronary artery calcification in chronic kidney disease (63)	- Ligand of farnesoid X receptor (64)
	Ursodeoxycholic acid	- Improving peripheral blood flow in chronic heart failure patients (65)	- Modulating potassium conductance (66) - Protection against reperfusion injury (PI3K/Akt pathway) (67)
	*Taurodeoxycholate*		
Short-chain fatty acid	Propionic acid	- Modulates blood pressure (68)	- Inhibition of cardiac hypertrophy, fibrosis, vascular dysfunction, and hypertension (in mice) (69)
	Butyric acid	- Associated with lower blood pressure (70)	- Protective effect against atherosclerosis (71) - Inhibition of histone deacetylase in cardiac hypertrophy (72) - Reduces fibrosis and induced angioneogenesis in myocardium (in mice) (72)
	Valeric acid	- Predictor of type II diabetes (73) - Negatively associated with insulin sensitivity, LDL and HDL (74)	- Reduction of blood pressure and heart rate (in rats) (75)

*Metabolites in italic: biological function is still unclear. CVD, cardiovascular diseases; ROS, reactive oxygen species; ACVD, atherosclerotic cardiovascular disease; MACE, major adverse cardiovascular event*.

### IPA Inhibits Cell Proliferation and Mitochondrial Function in HL-1 Cardiomyocytes

**Figure 2:**
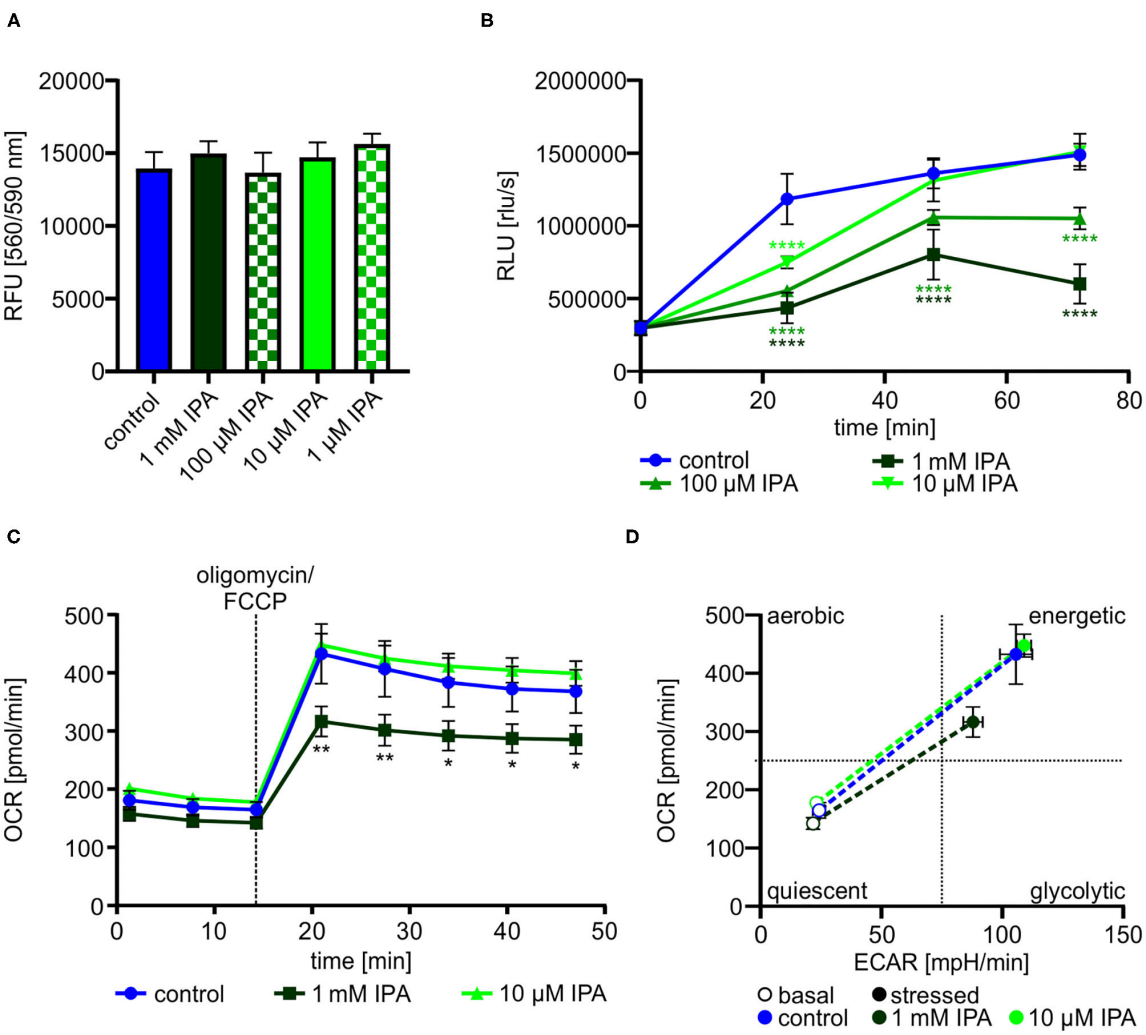
Effects of indole-3-propionic acid (IPA) on HL-1 cardiomyocytes. **(A)** Cell viability and cytotoxic effects of IPA after 24 h of treatment in HL-1 cardiomyocytes by resazurin-based assay. RFU, relative fluorescence units. *N* = 4/group, mean ± SD, 1-way ANOVA with *post-hoc* test. **(B)** Cell proliferation was measured by BrdU assay after 24, 48, and 72 h treatment with IPA. RLU, relative light units. *N* = 5/group, mean ± SEM, 1-way ANOVA with *post-hoc* test. **(C,D)** Measurement of the metabolic function by Seahorse Flux Analyzer in HL-1 cardiomyocytes after incubation with IPA for 24 h. **(C)** Changes in oxygen consumption rate (OCR) over time. *N* = 5/group. Two-way ANOVA with Dunnett *post-hoc* test. Data are mean ± SEM. **(D)** Metabolic phenotypes including OCR and extracellular acidification rate (ECAR) under basal (opened cycle) and stressed (filled cycle) conditions. Data were shown as mean ± SEM with *N* = 5/group. *p < 0.05, **p < 0.01, and ****p < 0.0001.

Dependent on the previous findings in our screening approach, we further explored the effects of IPA on HL-1 cardiomyocytes. Cell viability and cytotoxity of HL-1 cardiomyocytes was not affected by IPA at concentrations used from 1 μM to 1 mM (Figure 2A). Based on the reduced mitochondrial function under stress conditions, we determined the effect of IPA on cell proliferation. After 72 h, proliferation was decreased −59.6 ± 4.1% by 1 mM IPA (control: 1,487,379.6 rlu/s ± 34,096.4 vs. 1 mM IPA: 601,400.0 rlu/s ± 60,458.6; *p* < 0.0001), whereas no growth deprivation was observed at 10 μM IPA (Figure 2B).

We next performed a detailed analysis of IPA on mitochondrial respiration in HL-1 cardiomyocytes. As previously shown in our screening approach, 1 mM IPA after 24 h incubation showed a significant reduction of stressed OCR (control: 432.6 pmol/min ± 51.5 vs. 1 mM IPA: 316.3 pmol/min ± 26.1; *p* < 0.001) (Figure 2C). Basal OCR remained unchanged in response to any concentration of IPA (Figure 2C). In accordance to our findings of reduced proliferation by IPA, evaluation of the metabolic phenotype revealed a shift from an energetic to a more quiescent phenotype in response to 1 mM IPA (Figure 2D).

### IPA Affects Maximal Respiration and Respiratory Spare Capacity in Cardiomyocytes

**Figure 3:**
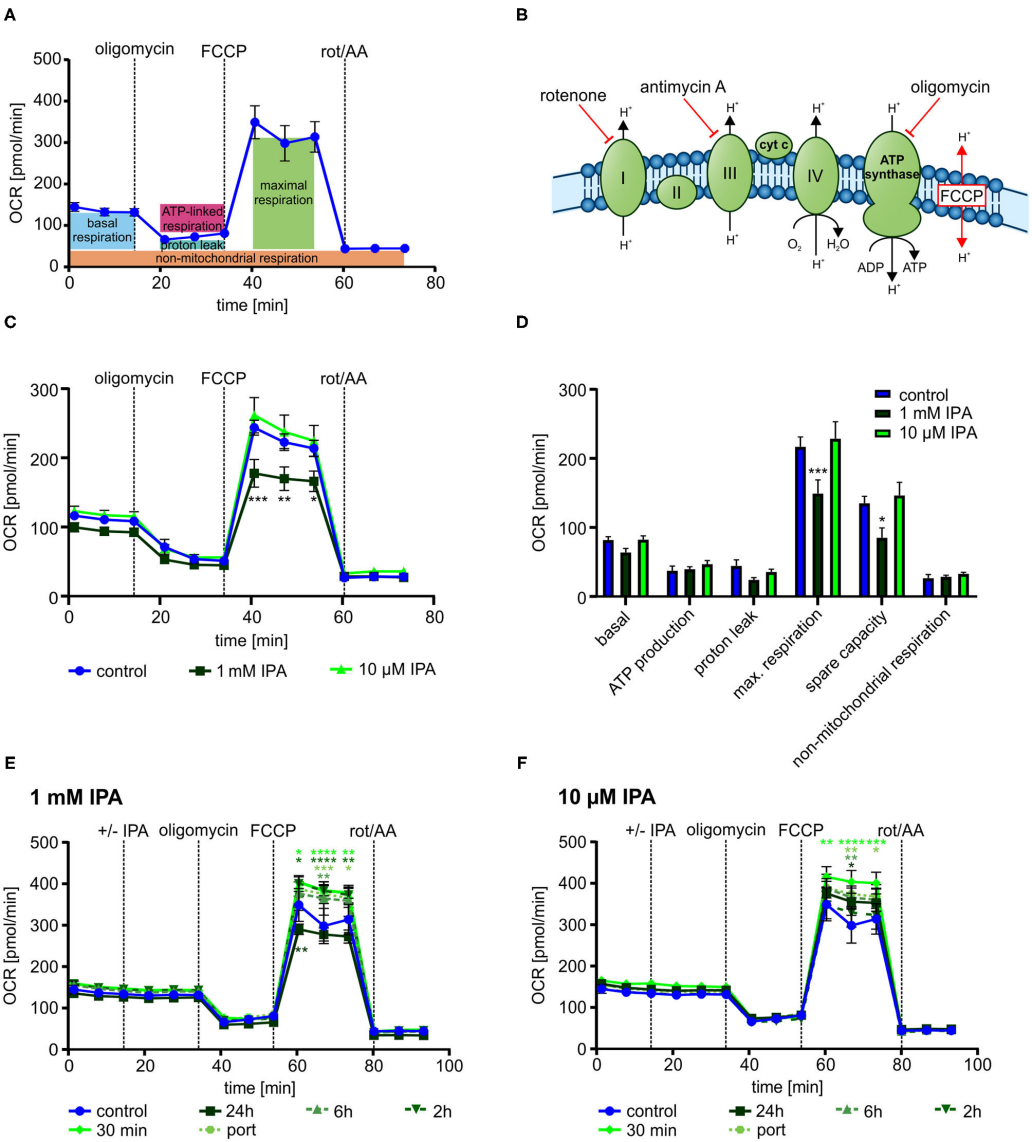
Mitochondrial characterization of HL-1 cardiomyocytes in response to treatment with indole-3-propionic acid (IPA). **(A)** Schematic representation of real-time measurement of mitochondrial function by Seahorse Flux Analyzer. Oligomycin, FCCP and rotenone/antimycin A (rot/AA) were injected sequentially with determination of basal respiration, ATP production, proton leak, maximum respiration including spare capacity and non-mitochondrial respiration. **(B)** Schematic overview on the mitochondrial respiratory chain with points of action of different inhibitors. **(C,D)** Mitochondrial stress testing of HL-1 cardiomyocytes after 24 h incubation with IPA. **(C)** Changes in oxygen consumption rate (OCR) over time in response to different inhibitors. Data were shown as mean ± SEM with *N* = 5/group. **(D)** Changes in basal respiration, ATP production, proton leak, maximal respiration, spare capacity, and non-mitochondrial respiration after 24 h incubation with IPA. For calculation, the last measurement points at basal and the first measurement points after injection of the respiratory chain inhibitors were used. Data were shown as mean ± SEM with *N* = 5/group. **(E,F)** Mitochondrial stress testing of HL-1 cardiomyocytes after incubation of IPA at different time points (24 h, 6 h, 2 h, 30 min and direct injection). **(E)** Changes in OCR over time in response to different inhibitors and IPA 1 mM. Data were shown as mean ± SEM with *N* = 5/group. **(F)** Changes in OCR over time in response to different inhibitors and IPA 10 μM. Data were shown as mean ± SEM with *N* = 5/group. For all data, *p < 0.05, **p < 0.01, ***p < 0.001, and ****p < 0.0001, 2-way ANOVA with Dunnett *post-hoc* test.

To gain deeper insights into the impact of IPA on mitochondrial respiration, we next evaluated mitochondrial function in response to serial injections different modulators of the respiratory chain (Figures 3A,B).

After 24 h of incubation, 1 mM IPA showed a significant decrease of OCR after FCCP injection in HL-1 cardiomyocytes (control: 243.5 pmol/min ± 10.8 vs. 1 mM IPA: 177.5 pmol/min ± 20.0; *p* < 0.001) (Figure 3C). However, no changes were observed at baseline as well as after injections of oligomycin and combination of rotenone/antimycin A, thus indicating a specific effect of IPA on maximal respiration (−18.9 ± 9.1%; *p* < 0.001) including respiratory spare capacity (−37.0 ± 10.6%; *p* < 0.05) (Figures 3C,D).

While the previous effects by IPA were observed after a 24 h incubation period, we next asked whether IPA exerts acute effects on mitochondrial function. Therefore, we set up an experiment with different incubation times of IPA. Cells were treated for 24 h, 6 h, 2 h, and 30 min with IPA prior to the assay or by direct port-based injection of IPA during the real-time assay. While the previously observed reduction of maximum respiration by 1 mM IPA after 24 h incubation was confirmed, we observed in both tested concentrations, 1 mM and 10 μM IPA, an increase of maximum respiration and spare capacity particularly at the shorter incubation times with a maximum at 30 min of incubation (Figures 3E,F). Remarkably, the greatest enhancement of maximal respiration of +21.5 ± 7.8% was observed at a concentration of 10 μM IPA after 30 min (control: 305.2 pmol/min ± 40.6 vs. 10 μM IPA: 370.8 pmol/min ± 23.7; *p* < 0.05). However, also an increase of OCR in response to FCCP was noticed in cells treated with direct injections of IPA during the assay (Figures 3E,F).

### IPA Exhibits Direct Effects on Cardiac Function in an Isolated Perfused Heart Model

**Figure 4:**
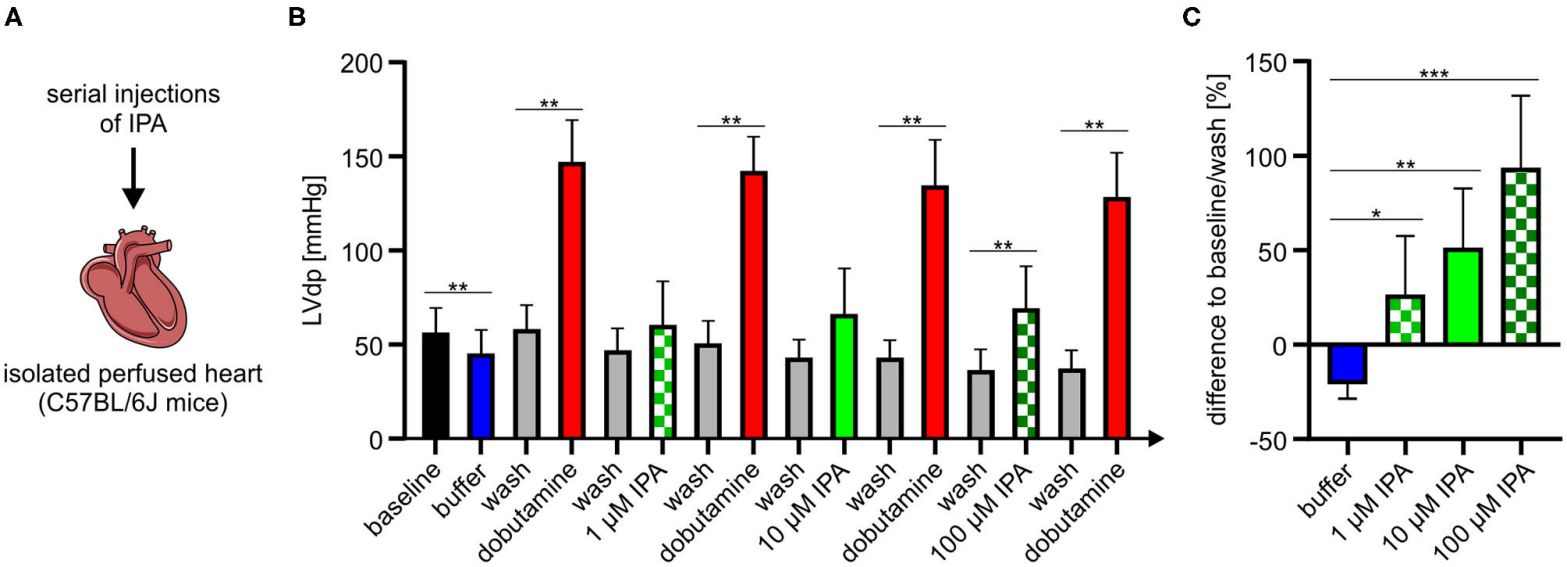
**(A)** Schematic overview of experimental procedure of Langendorff model. **(B)** Serial injections of buffer, dobutamine, and IPA with intermittent washing steps in the Langendorff mouse model show dose-depended effects of IPA on left ventricle developed pressure (LVdp). Dobutamine (1 mg/ml, 1:256) was administered as positive control. **(C)** Percentage difference of LVdp in relation to baseline/wash between buffer, IPA, and wash. All data were shown as mean ± SD with N = 7/group. *p < 0.05, **p < 0.01, and ***p < 0.001 by 1-way ANOVA.

Our previous experiments revealed that IPA modulates mitochondrial function in cardiac cells. Therefore, we asked whether IPA also affects cardiac function. An isolated perfused mouse heart model was used to evaluate the effects of IPA on heart function *ex vivo* (Figure 4A). In agreement with our previous findings of increased maximum respiration after short-term administration of IPA *in vitro*, we observed in the Langendorff model that IPA dose-dependently increased left ventricular developed pressure (LVdp), a validated parameter of cardiac function (Figures 4B,C). A percentage enhancement of LVdp from +26.4 ± 11.6% at 1 μM IPA (*p* < 0.05), +51.6 ± 11.7% at 10 μM (*p* < 0.001) and up to +93.6 ± 14.4% at 100 μM IPA (*p* < 0.0001) was observed. As expected, IPA also increased other cardiac performance indices such as LV pressure, dLVPmax, and dLVPmin (Supplementary Table 1). Serial injections of buffer did not have any effects on cardiac performance, e.g., shown by LVdp (Supplementary Figure 2).

### Impact of IPA on Mitochondrial Function in Human Hepatic and Endothelial Cells

**Figure 5:**
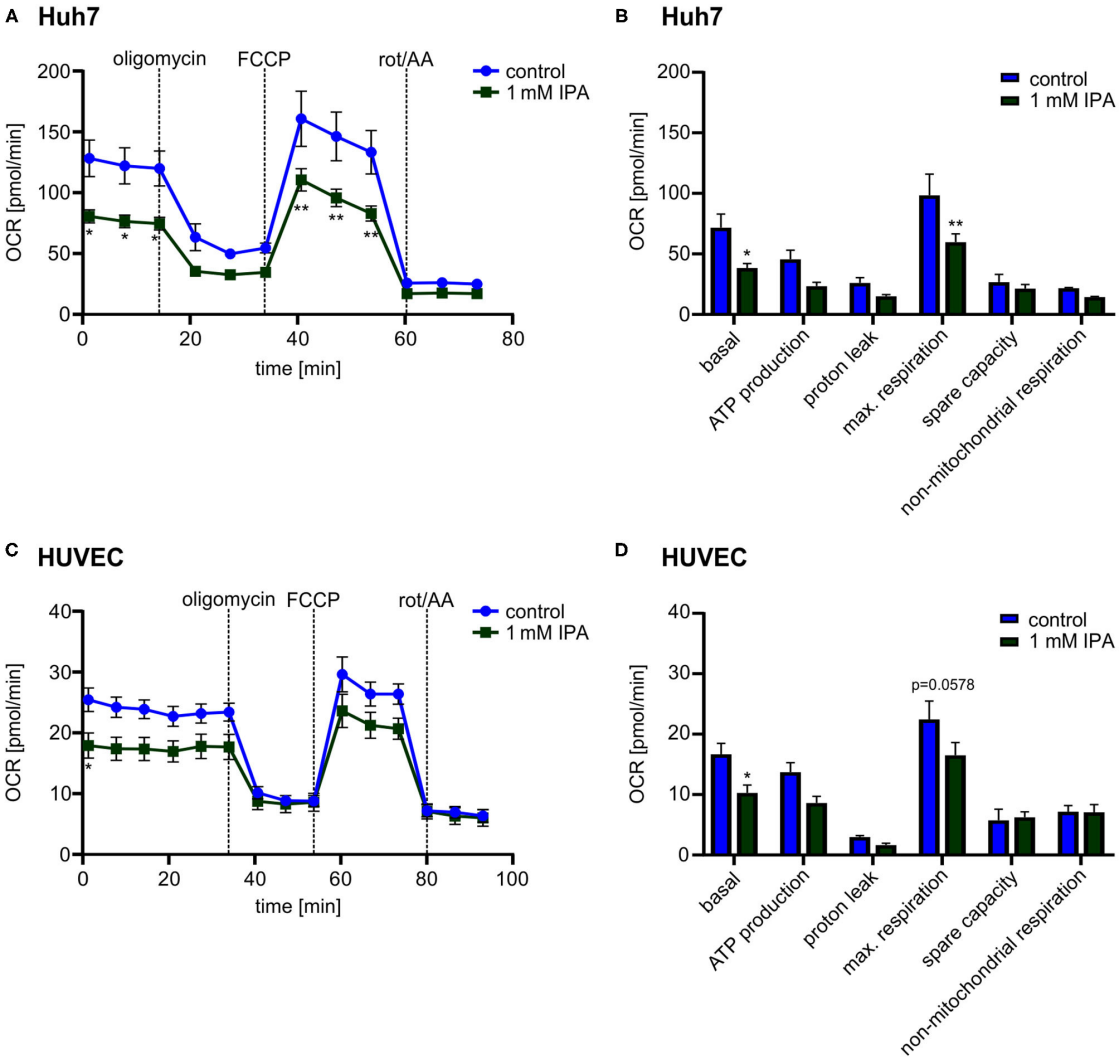
Mitochondrial characterization in human hepatic cell line (Huh7) and Human Umbilical Vein Endothelial Cells (HUVEC) in response to 1 mM indole-3-propionic acid (IPA) after 24 h. **(A,B)** Mitochondrial stress testing in Huh7 after 1 mM IPA treatment for 24 h. **(A)** Changes of oxygen consumption rate (OCR) over time in response to respiratory chain inhibitors. Data were shown as mean ± SEM with *N* = 5/group. **(B)** Changes in basal respiration, ATP production, proton leak, maximal respiration, spare capacity and non-mitochondrial respiration after 24 h incubation of 1 mM IPA. For calculation, the last measurement points at basal and the first measurement points after injection of the respiratory chain inhibitors were used. Data were shown as mean ± SEM with *N* = 5/group. **(C,D)** Mitochondrial stress testing in HUVEC after 1 mM IPA treatment for 24 h. **(C)** Changes of OCR over time in response to respiratory chain inhibitors. Data were shown as mean ± SEM with *N* = 5/group. **(D)** Changes in basal respiration, ATP production, proton leak, maximal respiration, spare capacity and non-mitochondrial respiration after 24 h incubation of 1 mM IPA. Data were shown as mean ± SEM with *N* = 5/group. Rot/AA, combination of the inhibitors rotenone and antimycin A. *p < 0.05 and **p < 0.01, 2-way ANOVA with Dunnett *post-hoc* test.

While the previous effects were observed in the murine heart, we wanted to know whether IPA affects mitochondrial function also in other tissues as well as in human cells. Therefore, we evaluated the effect of IPA in human hepatic cell line (Huh7) and Human Umbilical Vein Endothelial Cells (HUVEC). Both, Huh7 and HUVEC, showed a reduction of the maximal respiration after IPA treatment similar to the effects in HL-1 cardiomyocytes (Huh7: control: 98.4 pmol/min ± 17.6 vs. 1 mM IPA: 59.8 pmol/min ± 6.9; *p* < 0.01; HUVEC: control: 22.4 pmol/min ± 3.1 vs. 1 mM IPA: 16.5 pmol/min ± 2.1; *p* = 0.0575) (Figure 5). However, although toxicity of IPA was excluded in these cells, basal respiration (Huh7: −46.4 ± 5.0% and HUVEC: −38.5 ± 7.7%; both *p* < 0.05) was also significantly decreased (Figures 5B,D). Together, these findings indicate that effects of IPA on mitochondrial function is not specific for cardiac tissue and mouse cells.

### Evaluation of Mechanisms Associated Mitochondrial Modulation by IPA

**Figure 6:**
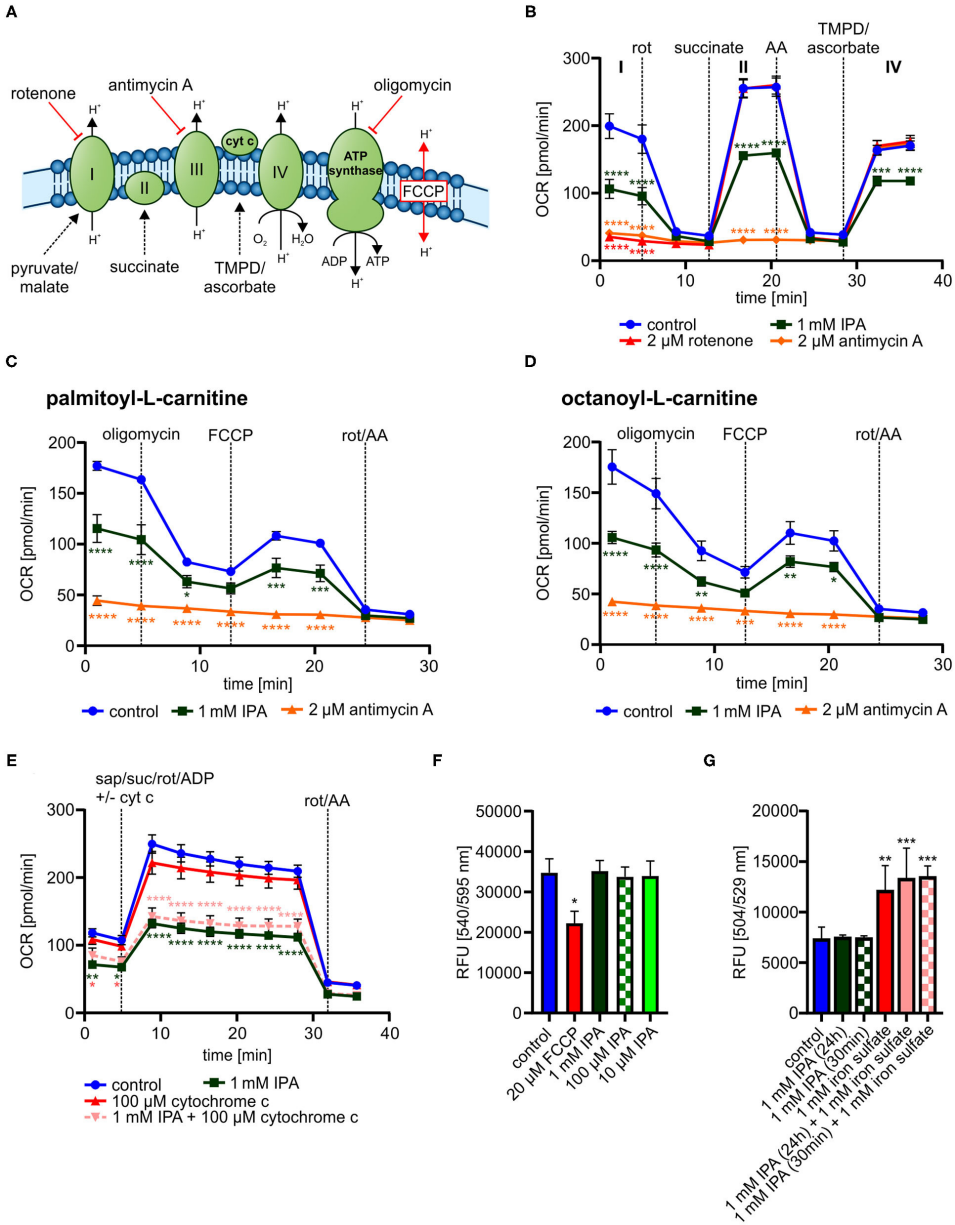
Detailed characterization of mitochondrial function in response to indole-3-propionic acid (IPA) in HL-1 cardiomyocytes. **(A)** Schematic representation of respiratory chain including substrates for detailed analysis of respiratory chain complexes. TMPD: *N,N,N*′*,N*′-Tetrametyhl-*p*-phenylenediamine. **(B)** Measurement of oxygen consumption rate (OCR) over time in response to substrates of respiratory chain complexes injected sequentially. Rot, rotenone; AA, antimycin A. **(C)** Mitochondrial stress testing in permeabilized HL-1 cardiomyocytes including palmitoyl-L-carnitine in response to 1 mM IPA (24 h). **(D)** Mitochondrial stress testing in permeabilized HL-1 cardiomyocytes including octanoyl-L-carnitine in response to 1 mM IPA (24 h). **(E)** Addition of exogenous cytochrome c to permeabilized HL-cardiomyocytes after treatment with IPA (24 h). Measurement of OCR over time. Sap, saponin; suc, succinate; rot, rotenone; cyt c, cytochrome c. **(B–E)** Data were shown as mean ± SEM with *N* = 5/group by 2-way ANOVA with Dunnett *post-hoc* test. **(F)** Mitochondrial membrane potential was measured by TMRE staining in HL-1 cardiomyocytes after treatment with IPA (24 h). 20 μM FCCP for 10 min was used as positive control. RFU: relative fluorescence units. Data were shown as mean ± SD with *N* = 4–5/group by 1-way ANOVA with *post-hoc* test. **(G)** Measurement of ROS production and hydroxyl radicals by H2DCFDA assay in HL-1 cardiomyocytes. HL-1 cardiomyocytes were treated with 1 mM IPA for 24 h and 30 min., 1 mM iron(II) sulfate for 30 min was used as positive control. Data were shown as mean ± SD with *N* = 4/group by 1-way ANOVA with *post-hoc* test. *p < 0.05, **p < 0.01, ***p < 0.001, and ****p < 0.0001.

We next explored mechanisms associated to modulated metabolic function by IPA. While a clear impact of IPA on mitochondrial respiration was observed, no effects of IPA on glycolysis were noticed (Supplementary Figure 3). These findings indicate that IPA may pre-dominantly modulate the mitochondrial respiratory chain. Therefore, single complexes of the respiratory chain were analyzed in the next steps (Figure 6A).

In all tested complexes, 1 mM IPA significantly reduced OCR approximately from 50 to 30% in a similar way (*p* < 0.001–0.0001) (Figure 6B), while rotenone (2 μM for 30 min) (complex I) and antimycin A (2 μM for 30 min) (complex II) did not show respiratory activity at the specific complex (Figure 6B). This finding indicates that IPA has no specific activity on complex I, II, or IV.

While the entire respiratory chain seemed to be affected by IPA without a complete blocking, we next tested whether IPA modulated mitochondrial fatty acid oxidation (FAO). FAO is an important energy source for mitochondrial respiration and defective FAO may eventually lead to mitochondrial dysfunction (21). In both analyzed FAO pathways, 1 mM IPA significantly reduced OCR (basal: palmitoyl-L-carnitine: −34.8 ± 7.8% and octanoyl-L-carnitine: −39.8 ± 3.5% (both *p* < 0.0001); max. respiration: palmitoyl-L-carnitine: −29.2 ± 8.9%; *p* < 0.001 and octanoyl-L-carnitine: −25.6 ± 5.2%; *p* < 0.01) (Figures 6C,D). As seen in our previous analysis of complex I, II, and IV, respiratory activity was reduced by IPA, but not completely inhibited as by the positive control antimycin A (Figures 6C,D).

While our previous data indicate modulation of FAO by IPA, involvement of cytochrome c release as well as changes in the mitochondrial membrane potential by IPA may also explain a general reduction of mitochondrial respiratory function. As shown in Figure 6E, cytochrome c supplementation was not able to restore the effect of IPA. Additionally, mitochondrial membrane potential was significantly affected by the positive control FCCP (20 μM for 10 min, *p* < 0.05), but no effect was observed by IPA (Figure 6F). Oxidative stress is another strong modulator of mitochondrial function. IPA has been described as antioxidant without pro-oxidative ability in neurons with pre-dominately protective effects against hydroxyl radicals (43, 76). Therefore, the impact of IPA on ROS production with focus on hydroxyl radicals was further analyzed. Neither alone nor in combination with the positive control iron(II) sulfate (1 mM), IPA modulated hydroxyl radical production in HL-1 cardiomyocytes (Figure 6G).

Independently of mitochondrial function, IPA has been described as agonist to human and rodent pregnane X receptor (PXR) (31). Therefore, we tested the effects of PXR agonists on mitochondrial function not only in murine HL-1 cardiomyocytes, but also in human Huh7 hepatic cells. Rifampicin was used as human PXR agonist and 5-Pregnen-3β-ol-20-one-16α-carbonitrile (PCN) as murine PXR agonist, as previously described (77, 78). In Huh7, 1 mM IPA and rifampicin revealed similar effects on OCR with no additional reduction by combination of both substances (control: 160.8 pmol/min ± 22.7; 1 mM IPA: 110.6 pmol/min ± 9.2; 10 μM rifampicin: 111.1 ± 18.5 pmol/min; 1 mM IPA and 10 μM rifampicin: 112.5 pmol/min ± 8.9; all *p* < 0.001) (Supplementary Figure 4A). While these results in Huh7 may indicate a PXR-dependent effect of IPA, murine PXR agonist PCN alone or in combination with IPA did not modify OCR in HL-1 cardiomyocytes (Supplementary Figure 4B). Neither did rifampicin (as weak agonist of murine PXR) modulate respiratory function in HL-1 cardiomyocytes (Supplementary Figure 4B). Together with the fact that PXR is only weakly expressed in the heart, a PXR-dependent effect of IPA on cardiac mitochondrial function seems unlikely.

## Discussion

Gut microbiota play an important role in the onset and progression of cardiometabolic diseases (79). The interaction between host and intestinal bacteria is complex and involves many different mechanisms. Particularly, these include microbiota dysbiosis (11, 80), modulation of energy harvest, induction of systemic low-grade inflammation, immunological reactions as well as microbiome-related metabolites (1, 81–83). Gut microbiota produce a vast number of metabolites, which enter blood stream and may exert biological functions in the host (1, 79). Several gut-derived metabolites have been identified to be associated with cardiovascular disease (CVD). While serum levels of TMAO have been linked to increased cardiovascular mortality and enhanced atherothrombotic risk (3, 4, 84), other gut-derived metabolites exert positive effects in the cardiovascular system. Particularly, SCFA have been described as positive modulators of CVD (71, 80, 85). Recently, we and others found beneficial effects of microbiota-derived tryptophan metabolites in relation to CVD (7, 83).

While many studies revealed the association between gut bacteria and cardiovascular diseases such as hypertension and atherosclerosis, little is known about the impact of the gut microbiome on heart failure (HF). Patients with HF show changes in their gut microbiome, but a direct link between onset of HF and microbiota could not been established so far (11). HF is a heterogenous disease with different pathomechanism involved. However, a molecular hallmark of HF is mitochondrial dysfunction in cardiomyocytes (12, 13). Metabolomics analyses have identified a large number of circulating metabolites that are derived from gut bacteria metabolism. To date, the impact of those metabolites on mitochondrial function in cardiomyocytes has not been evaluated. Therefore, we selected 25 different gut-derived metabolites with potential biological activity and screened their impact on metabolic function with focus on mitochondrial respiration in cardiomyocytes.

Unexpectedly, our screening approach did not discover many metabolites that affect metabolic function in cardiomyocytes. Choline derivatives and SCFA, substances that have been associated to CVD, did not modify metabolic activity in cardiomyocytes in our study. Beneficial effects of SCFA on mitochondrial function have been described in Akt2-knockout cardiomyocytes before, but no effects were detected in wildtype cardiomyocytes, as seen in our study (86). In a mouse model of myocardial infarction, administration of antibiotics decreased cardiac function by reducing levels of SCFA. The authors suggested a mechanism of modulation of myeloid cells by SCFA (80). Therefore, SCFA might not affect cardiomyocyte function. Biological effects of TMAO have been associated to atherosclerosis and atherothrombosis (4, 84). In agreement with these findings, TMAO has been predictive for mortality in ischemic HF, but not dilative HF or HF with preserved ejection fraction (5, 87). Thus, TMAO might not directly modulate mitochondrial function in cardiomyocytes.

However, we found that several gut-derived tryptophan derivatives, which exerted effects on respiration of cardiomyocytes under stress conditions. Of all analyzed metabolites, tryptophan derivative indole-3-propionic acid (IPA) showed the largest impact on stressed OCR. IPA is a circulating metabolite that is exclusively produced by gut bacteria, predominantly by *Clostridium sporogenes* (2, 88). Under physiological conditions, serum concentrations of IPA are around 1 μM in humans and 5 μM in rodents (40, 89). In contrast to other microbial tryptophan derivatives, like indoxyl sulfate or indole-3-acetic acid, IPA does not accumulate in chronic kidney disease (CKD) (35, 42, 90). Higher levels of IPA were even correlated with a lower risk of rapid renal decline in CKD patients (42). Effects of IPA on cardiometabolic diseases have been described as well. In human and mice, levels of IPA were negatively associated to the burden of atherosclerosis (7, 8). Further, low IPA levels were predictive for the incidence of type 2 diabetes mellitus (40, 41). In rodent models, administration of IPA reduced microbial dysbiosis and inflammation in steatohepatitis (91), reduced weight gain (89) and decreased inflammation in a model of colitis (31). Together, these studies indicate beneficial effects of IPA *in vivo*.

Based on the previous results, we investigated the effects of IPA on cardiomyocyte function more deeply. High, supraphysiological concentrations of IPA induced a more quiescent phenotype, which was linked to a specific reduction of maximal respiration and respiratory spare capacity in mitochondria. Reduced maximal respiration and spare capacity are both indicators of mitochondrial dysfunction. Spare capacity represent the reverse capacity of ATP, which can be used in response to cellular stress without a following ATP crisis (92). Mitochondria are the main cellular energy producers. In organs with high demands of energy, such as the heart, a defective mitochondrial respiration could be fatal. Mitochondrial dysfunction in cardiomyocytes is one of the first steps in molecular cardiac remodeling, eventually leading to clinical apparent HF (13, 15). In agreement with reduced mitochondrial function, we found that cardiomyocytes treated with IPA were less metabolic active revealing lower proliferation rates.

However, when we explored acute effects of IPA on mitochondrial respiration in a time series experiment, we observed the opposite effect of IPA on mitochondrial function. Both, low and high, concentrations of IPA increased maximal respiration with a maximum of 30 min after administration. This ambivalent characteristic of IPA leads to the speculation that mitochondrial capacity is first increased by IPA, but eventually induces mitochondrial damage by higher, supraphysiological concentrations of IPA in a later stage. However, 24 h treatment of high concentrations of IPA showed only reduced maximal respiration and spare capacity, but did not alter basal respiration or ATP production. Therefore, mitochondrial dysfunction of high-dose IPA might only apparent under cellular stress conditions.

The acute effects of IPA on mitochondrial function are in agreement with the observed effects of IPA on cardiac function in the isolated perfused mouse heart model. IPA immediately increased cardiac function, shown by increased contractility, already at low, physiological concentrations of IPA after acute administration. This effect might be explained by the acute modulation of mitochondrial function, as seen in our experiments with cardiomyocytes. While these data suggest positive cardiac effects of acute IPA treatment, further *in vivo* mouse experiments must address whether chronic administration of IPA exerts positive or negative effects on cardiac function and HF.

Based on these findings, we explored mechanisms that could be associated to modulation of mitochondria by IPA. IPA had no effect on glycolysis, thus indicating a pre-dominant role on mitochondrial respiration. Our experiments testing the impact of IPA on specific complexes of the respiratory chain did not point to a single complex. A possible target with impact on mitochondrial respiration, however, is oxidation of fatty acids by mitochondrial beta-oxidation. In cardiomyocytes, fatty acids are the pre-dominant fuel accounting for ~60–90% ATP production (15, 93). IPA equally reduced, but not completely inhibited long- and medium-chain fatty acid oxidation. This finding may indicate that IPA has an impact on fatty acid oxidation. Our experiments on cytochrome c involvement, mitochondrial membrane potential change or modulation of ROS production by IPA did not explain the observed mitochondrial effects of IPA. Therefore, modulation of FAO could also be a possible reason for a general decrease in respiration due to lower beta-oxidation. These data may also provide a possible explanation for reduced weight gain in rats upon treatment with IPA, as described by Konopelski et al. (89).

IPA has been described as antioxidant without pro-oxidative ability predominantly scavenging hydroxyl radicals in neurons (43, 76) and prevented against iron-induced oxidative damage in hepatic cells (44). Increase in reactive oxygen species (ROS) has closely been linked to mitochondrial dysfunction. However, we did not observe an effect of IPA on ROS production.

Our experiments in a human hepatic cell line as well as in primary human endothelial cells showed a similar decrease in mitochondrial respiration compared to our experiments in cardiomyocytes. Effects of IPA in other cell types have been described. The inhibitory effect of IPA on cell proliferation was, for example, observed in breast cancer cells, but mitochondrial function was not investigated (45). Besides an inhibition of maximal respiration, we observed in both cell types that also basal respiration was significantly reduced. This could indicate that these cell types are more sensitive to IPA compared to cardiomyocytes. A further explanation for this finding could be activation of pregnane X receptor (PXR) by IPA, as previously described (31, 45). PXR is a xenobiotic receptor, which is predominantly expressed in gastrointestinal and liver tissue, but has also been described in HUVEC before (94, 95). No or very low levels of PXR are expressed in cardiac tissue and HL-1 cardiomyocytes (94, 96). We observed that human PXR agonist rifampicin induced the same effect in Huh7 as IPA alone or in combination. These findings suggest that a reduction of mitochondrial respiration could also be caused by an activation of PXR due to IPA. However, rodent PXR agonist PCN did not show those effects on mitochondrial respiration in murine HL-1 cardiomyocytes. While in human hepatic cells our data are in agreement with PXR activation by IPA, the findings in cardiomyocyte make a general effect of IPA on mitochondria via PXR unlikely. An explanation for our results could be low expression of PXR in HL-1 cardiomyocytes, but also species differences among PXR activation in human and mice (97).

Our study has several limitations. In cardiovascular research, HL-1 cardiomyocytes are an established *in vitro* model to determine effects on cardiac tissue (96, 98). Therefore, we chose these murine instead of human or primary cardiomyocytes for our screening approach. Further, acute cardiac function was measured in isolated perfused hearts *ex vivo*. This model has the advantage to test direct cardiac effects of a substance without hormonal and neural influence. Nonetheless, *in vivo* studies are needed to verify our observed effects, especially to determine chronic effects of IPA on cardiovascular system. Acute and chronic administration of IPA in mouse models of HF, such as transverse aortic constriction and ligation of the left anterior descending artery, will allow to get deeper insights into the relationship between IPA and HF progression.

Mitochondrial dysfunction in our *in vitro* approach was observed only at supraphysiological conditions. However, stimulating effects of IPA on mitochondrial function in cardiomyocytes and cardiac function in the Langendorff model were observed at low, physiological concentrations (1–10 μM). Further, we could not fully elucidate the specific mechanism of IPA on mitochondrial function, although our data suggest involvement of FAO in HL-1 cardiomyocytes.

In conclusion, our cell-based screening approach identified that gut-derived metabolites are capable to affect cardiac physiology. Particularly, we could show that gut-derived tryptophan derivative IPA impacted mitochondrial respiration in cardiomyocytes as well as cardiac function in an isolated perfused heart model. Further studies will reveal whether IPA modulates cardiovascular function and HF *in vivo*.

## Data Availability Statement

The raw data supporting the conclusions of this article will be made available by the authors on reasonable request.

## Ethics Statement

The animal study was reviewed and approved by Government of North Rhine-Westphalia, Germany.

## Author Contributions

BK: conceptualization. MG and BK: methodology. MG and BK: development or design of methodology. MG: software. RS, KS, NM, and BK: formal analysis. MG, AN, and NK: investigation. KS, NM, and BK: resources. MG: data curation and writing–original draft. KS, RS, NM, and BK: writing–review and editing. MG, AN, and NK: visualization. RS, KS, NM, and BK: supervision. NM and BK: project administration. KS, NM, and BK: funding acquisition. All authors contributed to the article and approved the submitted version.

## Conflict of Interest

The authors declare that the research was conducted in the absence of any commercial or financial relationships that could be construed as a potential conflict of interest.
