# Supplementary material for: Gut-Derived Metabolite Indole-3-Propionic Acid Modulates Mitochondrial Function in Cardiomyocytes and Alters Cardiac Function
Source: Front Med (Lausanne). 2021 Mar 22;8:648259. doi: 10.3389/fmed.2021.648259 (PMC8019752; doi:10.3389/fmed.2021.648259)
Supplement: Supplementary file 1 [file Image_1.PDF]

Supplementary Figures

Supplementary Figure S1:

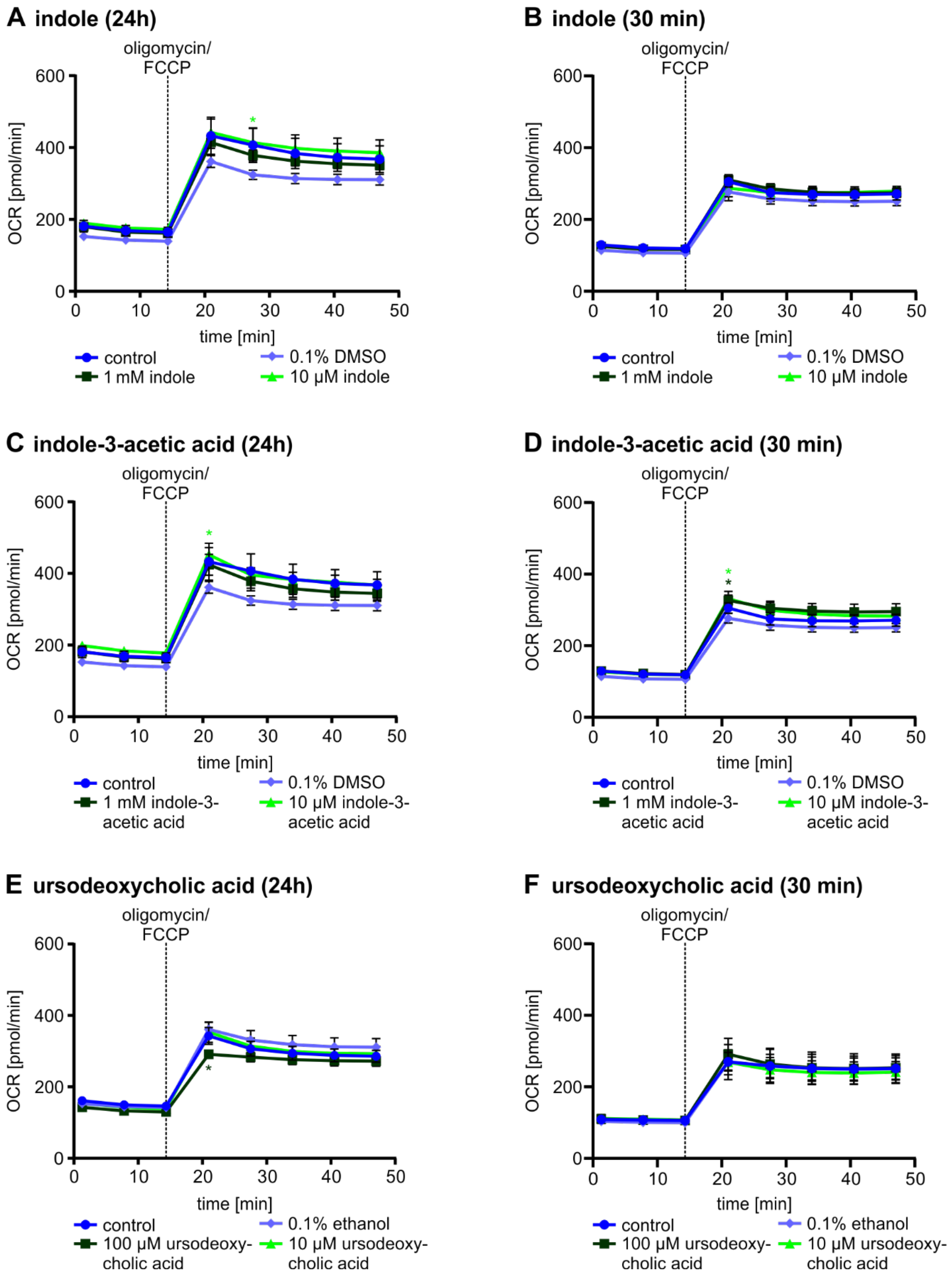

Supplementary Figure S1: Effects of chronic and acute treatment with indole, indole-3-acetic acid and ursodeoxycholic acid on mitochondrial respiration in HL-1 cardiomyocytes
(A) and **(B)** Measurement of the metabolic function by Seahorse Flux Analyzer in HL-1 cardiomyocytes after incubation with indole. **(A)** Changes in oxygen consumption rate (OCR) over time after treatment with indole for 24h. **(B)** Changes in OCR over time after an acute treatment for 30 min with indole. **(C)** and **(D)** Measurement of the metabolic function in HL-1 cardiomyocytes after indole-3-acetic acid treatment. **(C)** Changes in OCR over time after chronic treatment for 24h with indole-3-acetic acid. **(D)** Changes in OCR over time after treatment with indole-3-acetic acid for 30 min. **(E)** and **(F)** Measurement of the metabolic function in HL-1 cardiomyocytes after treatment with the bile acid ursodeoxycholic acid. **(E)** Changes in OCR over time after treatment with ursodeoxycholic acid for 24h. **(F)** Changes in OCR over time after treatment with ursodeoxycholic acid for 30 min. All data were shown as mean ± SEM with N=5/group, 2-way ANOVA with Dunnett post-hoc test. * p<0.05

Supplementary Figure S2:

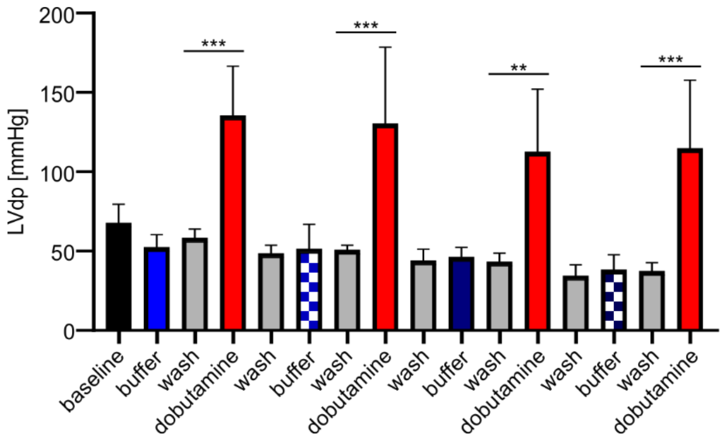

Supplementary Figure S2: Impact of serial buffer injections on *ex vivo* cardiac function in the isolated perfused Langendorff model
No significant effects of serial buffer injections were observed on left ventricle developed pressure (LVdp) in the Langendorff model. As positive control, dobutamine (1 mg/ml, 1:256) was administered. Data were shown as mean ± SD with N=4/group, 1-way ANOVA with Tukeys post hoc test. * p<0.05, ** p<0.01 and *** p<0.001.

Supplementary Figure S3:

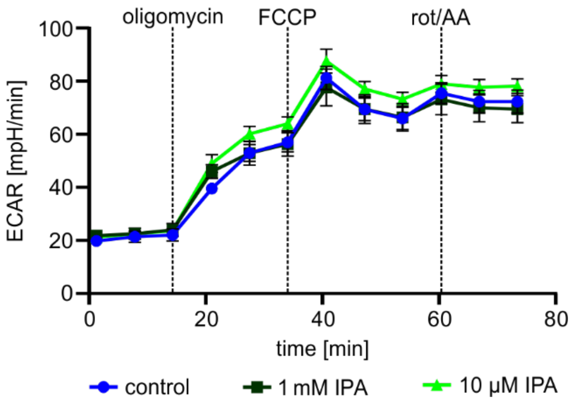

Supplementary Figure S3: Effects of indole-3-propionic acid (IPA) on glycolysis
Simultaneously measurement of extracellular acidification rate (ECAR) over time during mitochondrial stress testing by Seahorse Flux Analyzer. HL-1 cardiomyocytes were treated with IPA for 24h and response on glycolysis was measured after injection of respiratory chain inhibitors. No significant effect on glycolysis was observed at both tested concentrations of IPA. Rot/AA: mixture of the inhibitors rotenone and antimycin A. Data were shown as mean ± SEM with N=5/group, 2-way ANOVA with Dunnett post-hoc test.

Supplementary Figure S4:

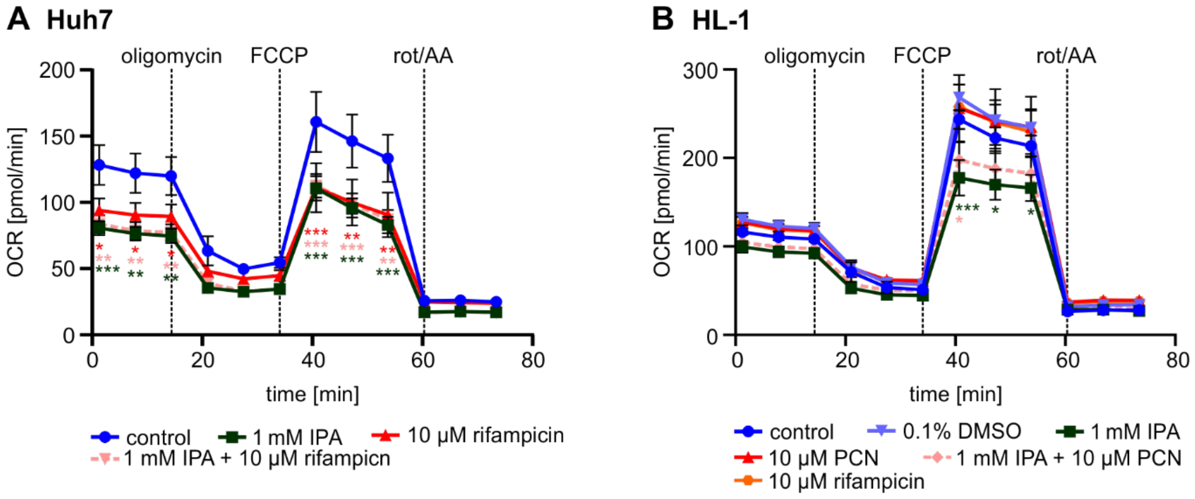

Supplementary Figure S4: Determination of pregnane X receptor (PXR)-related effects on Huh7 hepatic cells and HL-1 cardiomyocytes after indole-3-propionic acid (IPA) treatment

(A) Mitochondrial stress testing in Huh7 after treatment with 1 mM IPA for 24h. As positive control, human PXR agonist rifampicin for 24h was used. **(B)** Mitochondrial stress testing in HL-1 after treatment with 1 mM IPA for 24h. As positive controls, rodent PXR agonist 5-Pregnen-3β-ol-20-one-16α-carbonitrile (PCN) and rifampicin for 24h were used. Rot/AA: combination of rotenone and antimycin A. All data were shown as mean ± SEM with N=5/group. * $p<0.05$, ** $p<0.01$ and *** $p<0.001$ by 2-way ANOVA with Dunnett post-hoc test.
