# Supplementary material for: Gut-Derived Metabolite Indole-3-Propionic Acid Modulates Mitochondrial Function in Cardiomyocytes and Alters Cardiac Function
Source: Front Med (Lausanne). 2021 Mar 22;8:648259. doi: 10.3389/fmed.2021.648259 (PMC8019752; doi:10.3389/fmed.2021.648259)
Supplement: Supplementary file 2 [file Table_1.DOCX]

Supplementary Tables

## Supplementary Table 1:

| parameter | buffer | IPA 1 µM | IPA 10 µM | IPA 100 µM |
| --- | --- | --- | --- | --- |
| LVPsys | -10.83 ± 3.93 | 21.1 ± 24.4* | 37.25 ± 22.37** | 63.15 ± 26.29*** |
| LVPdia | 21.52 ± 11.61 | 3.37 ± 4.53*** | 2.21 ± 4.96*** | 4.2 ± 3.4*** |
| dLVPmin | -23.95 ± 8.52 | 21.26 ± 39.36 | 44.68 ± 41.36** | 75.56 ± 42.87*** |
| dLVPmax | -16.93 ± 3.76 | 24.87 ± 38.37 | 47.03 ± 44.13* | 72.35 ± 45.39*** |

**Supplementary Table 1:** Parameters of *ex vivo* cardiac function measured by the Langendorff model as difference in % from baseline/wash

Abbreviations: LVPsys: systolic left ventricular pressure; LVPdia: diastolic left ventricular pressure; dLVPmax: maximum rate of increase in left ventricular pressure during isovolumic contraction; dLVPmin: maximum rate of decrease in left ventricular pressure during isovolumic contraction. Data were shown as mean ± SD with N=7/group. * p<0.05, ** p<0.01 and *** p<0.001 by 1-way ANOVA with Tukeys post hoc test.
